# Terminal group effects of DOPO-conjugated flame retardant on polyamide 6: Thermal stability, flame retardancy and mechanical performances

**DOI:** 10.3389/fchem.2022.1002569

**Published:** 2022-09-28

**Authors:** Jing Gao, Wentao He, Yushu Xiang, Lijuan Long, Shuhao Qin

**Affiliations:** ^1^ Hubei Key Laboratory for Processing and Application of Catalytic Materials, Huanggang Normal University, Huangzhou, China; ^2^ National Engineering Research Center for Compounding and Modification of Polymer Materials, Guizhou, China

**Keywords:** terminal group effect, conjugated flame retardant, polyamide 6, flame retardancy, mechanical performance

## Abstract

Two DOPO-conjugated flame retardants with or without amino terminal groups (DOPO-NH_2_ and DIDOPO, respectively) were synthesized and incorporated into polyamide 6 (PA6). Results demonstrated the DOPO-NH_2_ endowed superior thermal, flame retardant and mechanical performances to PA6 composites. With the same loading of 15 wt%, DOPO-NH_2_ can catalyze the PA6 matrix more effectively and result in more residues at high temperature. The PA6 composites containing DOPO-NH_2_ exhibited higher LOI (28.0%) compared to 25.0% for the sample containing DIDOPO, and the lower heat release capacity and peak heat release rate. Furthermore, the overall mechanical properties of PA6 composites containing DOPO-NH_2_ outperformed the samples containing DIDOPO, even superior to that for PA6. Such a significant difference can be mainly attributed to the existence of amino-terminal group, which can interact with carboxyl group in PA6 as confirmed by dynamic mechanical analysis, improving the compatibility between the flame retardant and PA6 matrix.

## Introduction

As one class of important engineering plastics, polyamide especially polyamide 6 (PA6) are extensively used in electronical, automobile, and aerospace areas due to high tensile strength, chemical resistance, good electrical insulation and easy processability (Wirasaputra et al., 2016; Vannini et al., 2018; Song et al., 2017). However, the easy flammability greatly limits their application, and flame retardant modification of PA6 becomes an urgent task and is attracting more and more research interests.

Due to the environmental considerations, a variety of halogen-free flame retardants are proposed to flame retard PA6, including phosphorous-containing (Zhi et al., 2011; Tao et al., 2020), nitrogen-based (Cai et al., 2017; Lu et al., 2019), and some organic/inorganic flame retardants (Marneya et al., 2012; Zheng et al., 2020; He et al., 2020). Among these, metal phosphinates are proved to be the most efficient flame retardants for PA6 and has been commercialized as OP serials. Metal phosphinates play their main role in gas phase by releasing PO· free radical scavenger and also catalyze the charring in the condensed phase. Higher than 15 wt% loading of metal phosphinates is generally necessary to pass the UL-94 tests, which will inevitably cause loss in mechanical properties (Zhao et al., 2013; Lin et al., 2018). To further reinforce the flame retardancy and compensate the mechanical loss, inorganic synergists such as OMMT, carbon nanotube, halloysite are combined with metal phosphinates together to flame retard PA6 (He et al., 2017; He et al., 2019; He et al., 2022). In recent years, 9,10-dihydro-9-oxa-10-phosphaphenanthrene-10-oxide (DOPO) conjugated flame retardants have been developed as an effective alternative and exert their flame retardant activity mainly in gas phase (Xie et al., 2017; Koedel et al., 2020; Zhang et al., 2022; Sai et al., 2022). Incorporation of approx. 17 wt.% bridged DOPO derivatives (DiDopoMeo or DiDopoEDA) into PA6 can achieve a V-0 rating at 1 mm thickness (Buczko et al., 2014). In another work, a nitrogen-phosphorus-based DOPO derivative, DTE-DOPO was developed and incorporated to PA6. Compared to the commercially-available Exolit^®^ OP 1230, DTE-DOPO exhibited higher flame-retardant efficiency since lower P content was contained at a similar flame retardant loading, and superior mechanical performances (Butnaru et al., 2015). Therefore, DOPO derivatives exhibit good prospect in flame retarding PA6.

In our previous work, different DOPO derivatives were synthesized and employed to flame retard polylactic acid (PLA) and high-temperature PA (Long et al., 2017; Huang et al., 2018). Results demonstrated the molecular structure of DOPO derivatives played a critical role on the final flame retardant performance. For instance, the increase of aromatic ring in the structure of the flame retardant contributed to the formation of cross-linked structure in the residue of flame retardant polymers during combustion (Long et al., 2017). However, the effect of active terminal group in the molecules on the flame retardant efficiency have not been considered. Numerous researches have proved that active terminal groups such as epoxide or amino groups in flame retardants or flame retardant synergists have strong interaction with amino or carboxyl end-groups of polymer molecules by coupling reaction (Liang et al., 2019; Xu et al., 2019) or hydrogen bonding (Malkappa et al., 2020; Chen et al., 2017). The strong interaction between flame retardants and polymer matrix can improve the interfacial compatibility and promote the uniform dispersion of the flame retardants, which will be beneficial for improving the flame retardancy and thermomechanical or mechanical performance (Zhou et al., 2021b). For example, Malkappa et al. (Liu et al., 2021) found a 37.2% increase in thermomechanical performance and 41.7% and 30.4% decrease in the peak heat and total heat release rates, respectively, when adding 10 wt% poly (cyclotriphosphazene) functionalized α-zirconium phosphate (f-ZrP) nanoplatelets into PA6. Given that the possible strong interaction between −NH_2_ group of nanofiller and the carbonyl groups of PA6 polymer matrix, it is expected that the fire retardancy efficiency and mechanical performance of DOPO derivatives can be further improved by introducing −NH_2_ terminal group onto the molecular structure. Therefore, in this article, two DOPO-conjugated flame retardants with similar structures but different terminal groups were incorporated into PA6 to investigate the effect of terminal groups. The thermal stability and flame retardancy were investigated by thermogravimetric analysis (TGA), limited oxygen index (LOI) and microscale combustion calorimetry (MCC). The mechanical performances were evaluated by dynamical rheological analysis (DMA) and tensile testing.

## Materials and methods

### Materials

**FIGURE 1:**
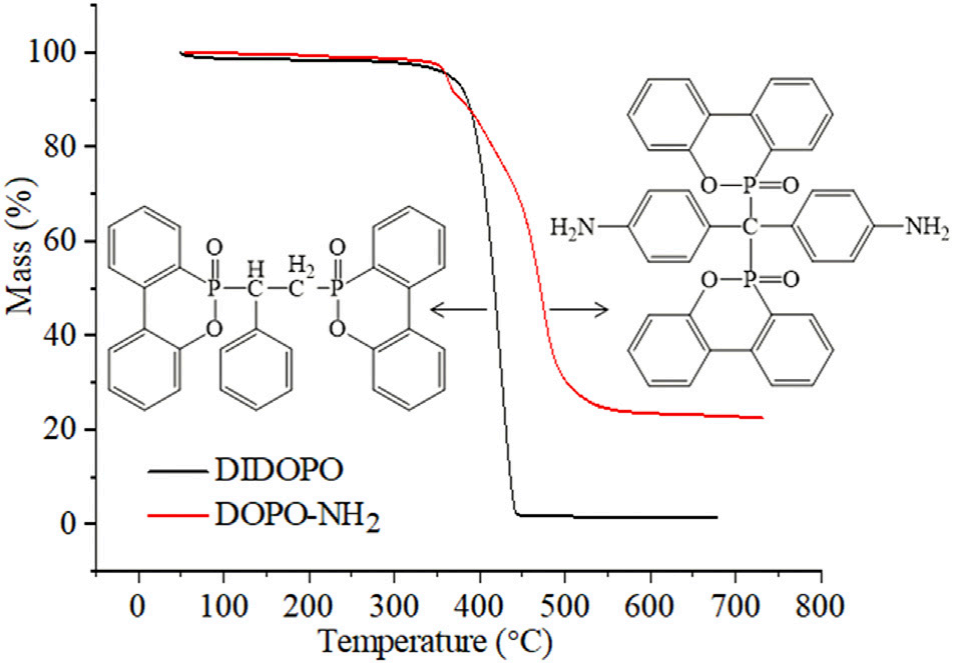
TG curves of DIDOPO and DOPO-NH_2_ under N_2_.

PA6 (1013B) were supplied by UBE Co. Ltd (Tokyo, Japan). The DOPO-based flame retardant DIDOPO without amino terminal groups (as shown in Figure 1), was synthesized as previously reported protocols (Long et al., 2017). Briefly, 86.4 g DOPO and 24 g 2-acetonaphthone were dissolved in xylene at 170°C. Then 16 g phosphorus oxychloride was added dropwise to the above solution. After reacting for 8 h, 100 ml isopropyl alcohol was added to the mixture and stirred at 80°C. The obtained solid product was then recrystallized, and washed with isopropyl alcohol repeatedly to obtain the final powder product. ^1^H NMR (CDCl_3_): δ 6.9–8.0 (m, 21H), 3.2 (m, 1H), 2.4 (m, 2H). HRMS (m/z): [MH]^+^ calcd for C_32_H_24_O_4_P_2_ 535. Found: 535.

The DOPO-NH_2_ with amino terminal groups was synthesized according to our previous work (Wang et al., 2021). Briefly, 38.91 g DOPO and 6.37 g 4, 4′-diaminobenzophenone were mixed. The mixture was then heated to 180°C and stirred for 3 h. After cooling down to 100°C, 150 ml toluene was added into the flask under stirring. The formed precipitate was filtered off and washed with toluene. The obtained solid product was then recrystallized from tetrahydrofuran and the final powder product was obtained. ^1^H NMR (DMSO-d6): δ5.82–8.06 (m, 24H), 4.92 (s, 4H). HRMS (m/z): [MH]^+^ calcd for C_37_H_28_O_4_N_2_P_2_ 627. Found: 627.

The flame retardant PA6 composites consisting of 85 wt% PA6 and 15 wt% flame retardants were prepared by melt compounding in a co-rotating twin screw extruder (CTE 20, Coperion Keya Machinery Manufacturing Co., Ltd., China) at about 210–230°C with the screw speed of 300 r/min and feeding speed of 16 r/min. Then, the modified PA6 composites were further injection-molded into the standard testing bars by an injection molding machine (CJ80MZ2NCII, Zhende Plastic Machinery Factory, China) at 210–230°C. The obtained PA6 composites were designated as PA6/DIDOPO and PA6/DOPO-NH_2_, respectively.

### Characterization

Thermogravimetric analysis (TGA) was carried out by a Q50 instrument (TA Instruments, United States) under nitrogen flow, from ambient temperature to 800°C with a heating rate of 10°C·min^−1^.

Crystallization and melting behavior were examined using a Q10 differential scanning calorimetry (DSC) (TA Instruments, United States). Samples of about 5–10 mg were heated from 40 to 250°C and held at 250°C for 5 min to erase the thermal history. Then, the samples were cooled to 40°C at a cooling rate of 10°C·min^−1^. A subsequent heating scan was then recorded at a rate of 10°C·min^−1^ from 40 to 250°C. The crystallinity was calculated according to the following equation:

$$Xc\left(\%\right)=\frac{\Delta Hm}{\Delta H0m\left(1-\Phi \right)}$$ (1)

where *Xc* is the crystallinity; Δ*Hm* is the melting enthalpy of the sample in the second heating; Δ*H0m* is the melting enthalpy of 100% crystalline PA6, which is 190 J/g (Xiao et al., 2017); *ϕ* is the mass fraction of the flame retardants in the composites.

The LOI test was conducted on a JF-3 oxygen index meter (Jiangning Analytical Instrument Company, Jiangning, China) with sample dimension of 100 mm × 6.5 mm × 3.2 mm according to the standard of ASTM D286377. Microscale combustion calorimetry (MCC) was carried out on a FTT0001 (Fire Testing Technology Ltd., United Kingdom) to determine the flammability properties of the PA6 composites of milligram-sized samples according to ASTM Standard Method D7309. The specimens were thermally decomposed in an oxygenated environment with a heating rate of 1 K s^−1^.

The rheological behaviors of samples were investigated by using a rheometric analyzer (HAAKE MARSII, Thermo Fisher Scientifc Inc., Newington, Germany) with the diameter of parallel plates 35 mm. The tests were carried out over an angular frequency range of 0.1 to 100 rad s^−1^ at 175°C using 1% strain. Tensile and flexural strengths of the samples were evaluated using a universal test machine (CMT4104, Shenzhen SANS Testing Machine Co., China) with cross-head speeds of 50 and 2 mm min^−1^ at room temperature. The notched Izod impact strength was measured according to ASTMD256A using an impact tester ZBC 1400-2. The freeze-fractured surfaces of flame retardant PA6 composites were recorded on a TESCAN MIRA scanning electron microscope (SEM) using 20 kV accelerating voltage. Prior to the measurements, the freeze-fractured surfaces were fractured and sputter-coated with 5 nm gold.

## Results and discussion

### Thermal stability of polyamide 6/DOPO-conjugated flame retardant composites

**FIGURE 2:**
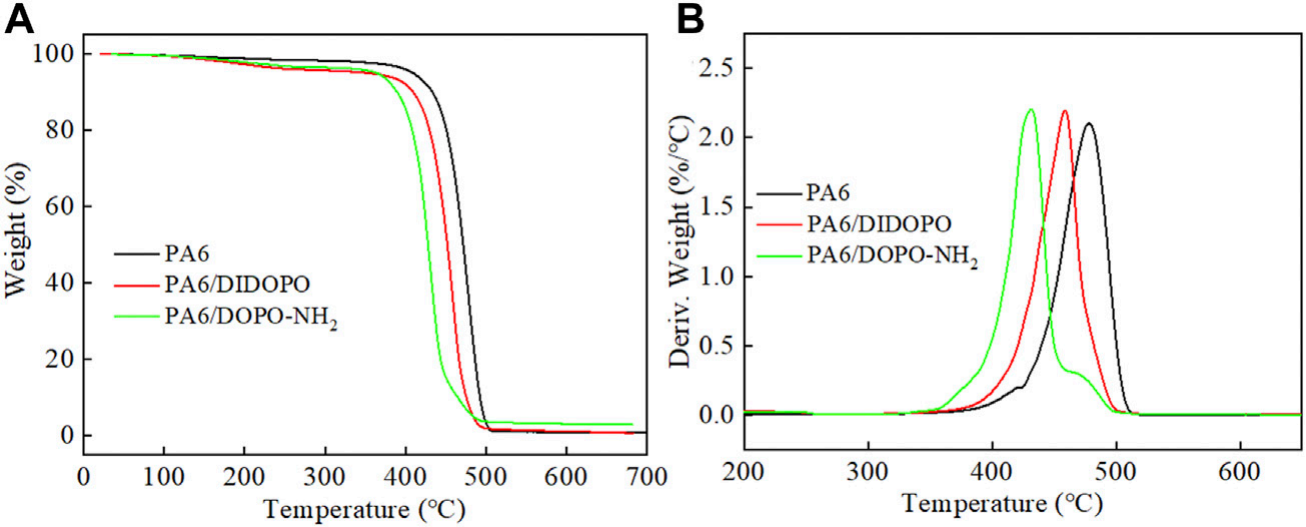
TG **(A)** and DTG **(B)** curves of PLA and flame retardant PLA composites under N_2_.

According to our previous work, the initial decomposition temperature (T_5%_) of DOPO is estimated at 249°C, and maximum decomposition temperatures (T_max_) is at 328°C (Wang et al., 2021). From Figure 1 and Table 1, both flame retardants exhibit better thermal stability compared to DOPO. The T_5%_ of DIDOPO under N_2_ is observed at 363.6°C. The compound decomposes in a single decomposition step with T_max_ at 427.3°C and the residues at 650°C is 1.5%. For DOPO-NH_2_, the T_5%_ and T_max_ is 361.2°C and 473.3°C, respectively. Different from DIDOPO, the char yield of DOPO-NH_2_ at 600°C reaches 23.5%, suggesting the better charring capacity. The TG curves of pure PA6 and PA6 composites in N_2_ atmosphere are revealed in Figure 2, and the typical data are listed in Table 1. Pure PA6 decomposes in one step process, with the T_5%_ and T_max_ at 408.6°C and 478.4°C, respectively, leaving negligible char residues. With the introduction of DIDOPO, the T_5%_ and T_max_ decrease and the amounts of char residues increase slightly. For PA6/DOPO-NH_2_, the amounts of char residues increase more significantly, indicating that DOPO-NH_2_ with amino terminal groups improves the thermal stability of the char residue at high temperature and promotes the formation of more residues (Zhou et al., 2019).

**TABLE 1:** TG and DTG data of flame retardants, PA6 and PA6 composites in N_2_.

Sample	T_5wt%_ (^o^C)	T_max_ (^o^C)	Residues (wt%)
DIDOPO	363.6	427.3	1.5
DOPO-NH_2_	361.2	473.3	23.5
PA6	408.6	478.4	0.9
PA6/DIDOPO	349.4	458.8	1.1
PA6/DOPO-NH_2_	363.5	431.5	3.1

### Differential scanning calorimetry analysis of polyamide 6/DOPO-conjugated flame retardant composites

**FIGURE 3:**
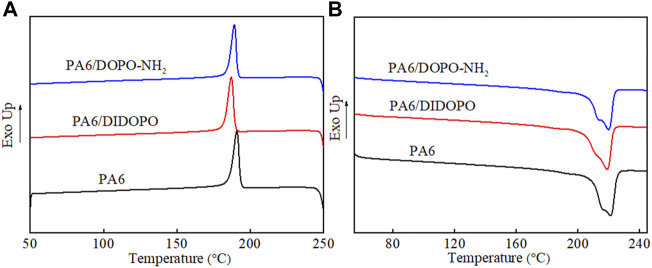
Nonisothermal crystallization **(A)** and melting **(B)** curves of PA6 and PA6 composites.

DSC analysis was conducted to investigate the effect of two flame retardants on the crystallization and melting behaviors of PA6. To erase the previous thermal history of the samples, an initial heating scan was performed. Then the cooling and second heating processes were recorded. As revealed by Figure 3A and Table 2, the crystallization temperature (T_c_) of the neat PA6 is located at 190.8°C during the cooling process. From Figure 3B, the melting curve of PA6 presents two peaks where the low temperature peak corresponds to the γ-crystalline form and the high-temperature peak corresponds to the α-crystalline form (Cai et al., 2017). With the incorporation of DIDOPO, the crystallization temperature reduces by about 3°C (187.0°C) and lower melting temperatures (T_m_) are observed. Similar decrease in melting temperatures for polymer composites by other DOPO derivatives is previously reported (Butnaru et al., 2015; Jia et al., 2018). The incorporated DIDOPO which acts as a plasticizer is regarded as the main cause. However, the T_c_ and T_m_ of PA6/DOPO-NH_2_ are all higher compared to PA6/DIDOPO. Besides, upon the addition of DIDOPO, the crystallinity (X_c_) of PA6/DIDOPO increases compared to the neat PA6 (from 31.8% to 34.7%), while the crystallinity of PA6/DOPO-NH_2_ is 31.7%. It is considered that for PA6/DIDOPO, the DIDOPO in PA6 acts as a plasticizer and facilitates the movement of polymer molecular chain segments, resulting in the higher crystallinity. While for PA6/DOPO-NH_2,_ the terminal amino groups in DOPO-NH_2_ can form hydrogen bonding with the PA6 matrix and therefore limit the crystallization process (Wang et al., 2019).

**TABLE 2:** Parameters of the nonisothermal crystallization and melting for PA6 and PA6 composites.

Sample	T_c_ (^o^C)	H_m_ (J/g)	X_c_ (%)	T_m_ (^o^C)
PA6	190.8	76.3	31.8	214.4/221.1
PA6/DIDOPO	187.0	70.8	34.7	209.3/218.8
PA6/DOPO-NH_2_	189.1	64.6	31.7	212.0/219.8

### Flame retardancy of polyamide 6/DOPO-conjugated flame retardant composites

The flame retardancy of PA6 and PA6 composites is investigated by LOI tests, and the results are listed in Table 3. PA6 is inherently flammable and the LOI value of 22.0% is obtained. The introduction of DIDOPO and DOPO-NH_2_ improves the LOI to 25.0% and 28.0%, respectively. Such results indicate that both flame retardants endow PA6 with improved flame retardancy and the flame retardant efficiency of DOPO-NH_2_ is superior to that of DIDOPO.

**TABLE 3:** The parameters obtained from MCC and LOI values for PA6 and PA6 composites.

Sample	HRC (J/g·K)	PHRR (W/g)	T_M_ (^o^C)	LOI (%)
PA6	693	683.1	489.9	22.0
PA6/DIDOPO	732	714.7	445.6	25.0
PA6/DOPO-NH_2_	675	670.5	467.3	28.0

**FIGURE 4:**
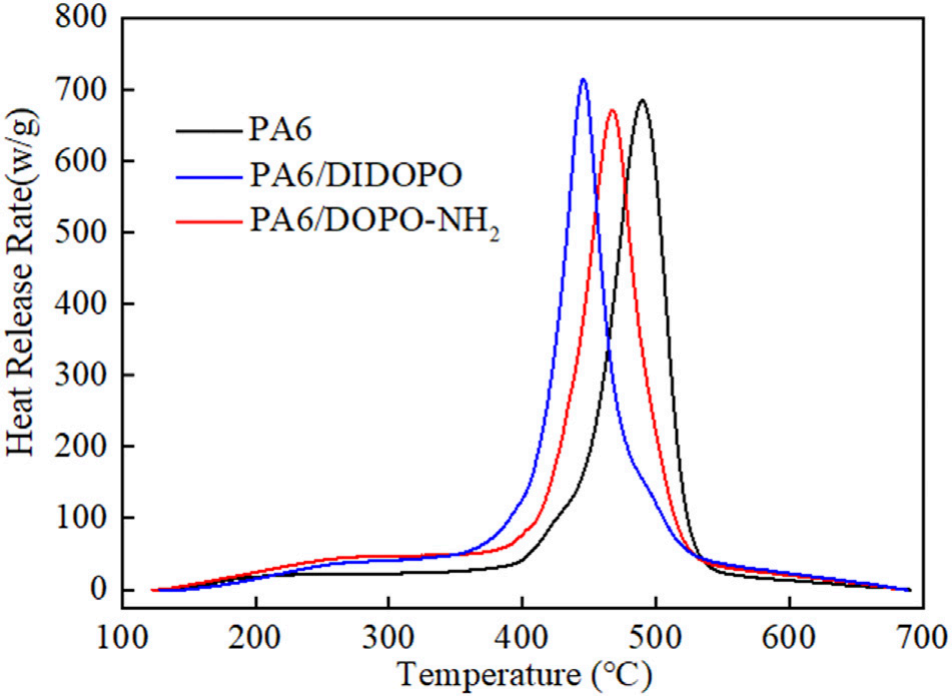
The HRR curves of PA6 and PA6 composites from MCC.

Microscale combustion calorimetry (MCC) is one of the most effective bench scale methods to evaluate the combustion properties of polymer materials of milligram-sized samples (Ding et al., 2016). In this work, MCC was employed in order to assess and compare the combustion behavior of different PA6 composites. The heat release rate (HRR) profiles of the PA6 and PA6 composites versus temperature are shown in Figure 4 and the relevant MCC data are listed in Table 3. For neat PA6, the peak heat release rate (pHRR) was 683.1 W g^−1^ at a temperature of T_M_ = 489.9°C and the heat release capacity (HRC) was about 693 kJ g^−1^. The presence of DIDOPO in the PA6 causes decrease in T_M_ due to the thermal degradation of DIDOPO or their catalytic degradation effect on PA6 as reflected by the slightly increased residues in TG analysis. In addition, the introduction of DIDOPO results in slightly increased PHRR and HRC (714.7 W g^−1^ and 732 kJ g^−1^, respectively). According to references (Buczko et al., 2014; Butnaru et al., 2015), similar phenomenon regarding the increased pHRR for the PA6 formulation containing DiDopoEDA or DTE-DOPO have been reported. It is considered that for these DOPO derivatives, a gas-phase flame inhibition mechanism plays a main role in improving the flame retardancy (Peng et al., 2021; Yang et al., 2021; Huo et al., 2022). On the contrary, the addition of DOPO-NH_2_ bring about decrease in PHRR and HRC (670.5 W g^−1^ and 675 kJ g^−1^), respectively. Therefore, in the present study, it can be concluded that for these DOPO derivatives, a gas-phase flame inhibition mechanism plays a main role in improving the flame retardancy. While for DOPO-NH_2_ containing sample, besides the flame inhibition in gas phase due to the existence of DOPO groups, the charring effect in condensed should not be excluded (Xue et al., 2021).

### Rheological and mechanical properties

**FIGURE 5:**
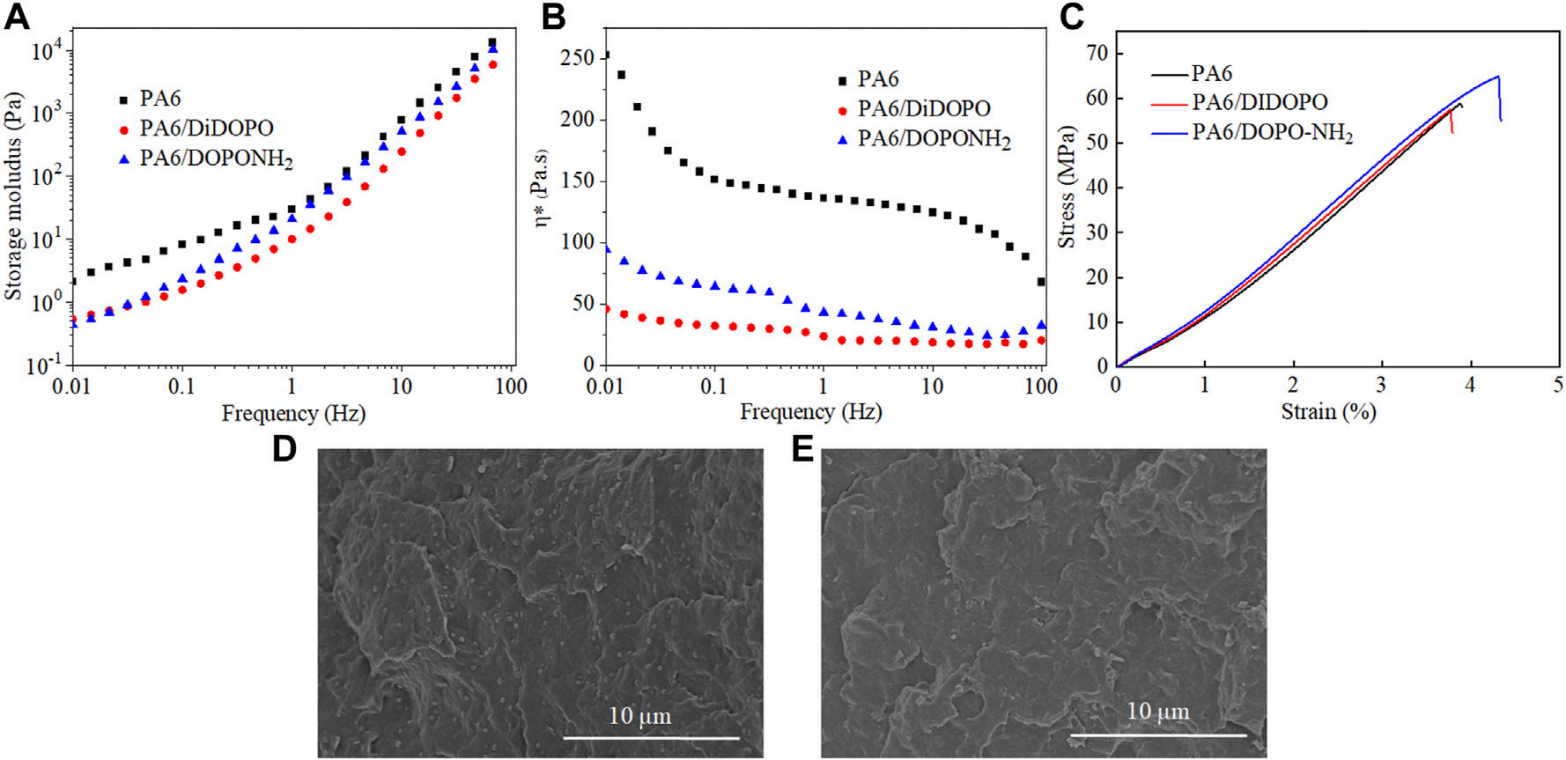
Storage modulus **(A)** and complex viscosity **(B)** as a function of frequency, and stress-strain curves **(C)** of PA6 and PA6 composites; SEM images of the fracture of PA6/DIDOPO **(D)** and PA6/DOPO-NH_2_ **(E)** composites after their treatment in liquid N_2_.

To evaluate the influence of the two flame retardants on the rheological behaviors of PA6, rheological measurements were carried out on neat PA6 and PA6 composites. From Figures 5A,B, both storage modulus and complex viscosity of PA6 decrease obviously with the incorporation of DiDOPO in the whole range of measured frequencies. This might indicate the internal plasticizing effect of DiDOPO on the polymer structures, which facilitates the movement of the PA6 molecular chains. For DOPO-NH_2_ containing samples, although the storage modulus and the complex viscosity decrease compared to neat PA6, the effect is not so profound. In almost the whole frequency range, the storage modulus and complex viscosity of PA6/DOPO-NH_2_ are higher than that of PA6/DIDOPO. This could be ascribed to the limited motion of the polymer chains due to the interaction between the terminal amino of DOPO-NH_2_ and carboxyl end groups of PA6 molecules (Wang et al., 2019).

Generally, the mechanical performance of polymer composites are strongly dependent on the interfacial compatibility between the additive and the polymer matrix (Qian et al., 2017; Qian et al., 2019; Liu et al., 2022). The impacts of DIDOPO and DOPO-NH_2_ on the mechanical properties PA6 matrix are assessed and the results are presented in Figure 5C and Table 4. Upon the loading of DIDOPO, the tensile and flexural strengths of PA6 decrease to 53.2 and 67.8 MPa, respectively, from 58.4 to 71.2 MPa for neat PA6. The elongation at break shows no obvious change. Comparably, in the presence of DOPO-NH_2_, the tensile and flexural strengths of PA6 composites increase to 63.7 and 81.3 MPa, respectively, even higher than that of untreated PA6. Besides, the strain at break further increased to 4.3% (higher than 3.9% of the untreated PA6).

**TABLE 4:** Mechanical properties of pure PA6 and PA6 composites.

Sample	Tensile strength (MPa)	Flexural strength (MPa)	Notched impact strength (kJ/m^2^)	Elongation at break (MPa)
PA6	58.4 ± 0.6	71.2 ± 3.0	9.2 ± 0.3	3.9 ± 0.1
PA6/DIDOPO	53.2 ± 4.1	67.8 ± 4.1	3.5 ± 0.1	3.8 ± 0.3
PA6/DOPO-NH_2_	63.7 ± 1.9	81.3 ± 2.1	3.6 ± 0.1	4.3 ± 0.3

In addition, PA6/DIDOPO and PA6/DOPO-NH_2_ composites registered lower impact strength values of 3.5 kJ/m^2^ and 3.6 kJ/m^2^, respectively, compared to pristine PA6, suggesting the incorporation of both flame retardants increases the brittleness due to its rigid chemical structure (Zhou et al., 2021a). From SEM images the cryogenic fracture of both PA6 composites, one can distinguish distinct lines of fracture propagation which are associated to an inherent brittleness Figures 5D,E. Similar phenomenon have been observed for PA6/meltable triazine-DOPO by Butnaru et al. (2015). It is considered that a good interfacial compatibility between the flame retardant and PA6 restricts the movement of the chain segments, resulting in a lower toughness. In addition, a few DIDOPO aggregate particles are clearly visible in the fracture surface in PA6/DIDOPO composites but not in PA6/DOPO-NH_2_ (Figures 5D,E), which can explain why the latter exhibits higher tensile strength and flexural strength than the former.

The results are consistent with the rheological analysis and it is considered that the terminal amino groups play a critical role, which can form strong hydrogen bonding with PA6 molecular chains and enhance the interaction force between PA6 macromolecular chains (Liu et al., 2021). However, the introduced flame retardant particles may act as an energy concentration under impact force, resulting in decrease of impact strength.

## Conclusion

In this paper, DIDOPO and DOPO-NH_2_ with amino terminal groups were used to modify the flame retardancy and mechanical properties of PA6. The flame retardant performance of PA6 containing DOPO-NH_2_ is superior to that containing DIDOPO. At 5 wt% loading of the additives, the PA6/DOPO-NH_2_ had a LOI of 28.0% while PA6/DIDOPO show a much lower LOI of 25.0%. Besides, from the MCC test, the PHRR and HRC of PA6/DOPO-NH_2_ decreased compared to that of pristine PA6, and the PHRR and HRC of PA6/DIDOPO are even higher than of PA6. Furthermore, the overall mechanical performances of PA6 containing DOPO-NH_2_ are better than the sample containing DIDOPO. Particularly, the tensile strength, flexural strength and elongation at break of PA6 composites containing DOPO-NH_2_ reach 63.7, 81.3 MPa and 4.3%, respectively, even higher than that of untreated PA6. DSC and rheological analysis suggest that the stronger interaction between DOPO-NH_2_ and PA6 play a critical role for the property improvement. This work offers an alternative and promising strategy for design and synthesis of more efficient flame retardants.

## Data Availability

The original contributions presented in the study are included in the article/Supplementary Material, further inquiries can be directed to the corresponding author.
